# Network-Based Methods for Approaching Human Pathologies from a Phenotypic Point of View

**DOI:** 10.3390/genes13061081

**Published:** 2022-06-17

**Authors:** Juan A. G. Ranea, James Perkins, Mónica Chagoyen, Elena Díaz-Santiago, Florencio Pazos

**Affiliations:** 1 Department of Molecular Biology and Biochemistry, University of Malaga, 29071 Malaga, Spain; ranea@uma.es (J.A.G.R.); jimrperkins@gmail.com (J.P.); elenadiazsantiago.92@gmail.com (E.D.-S.); 2 CIBER de Enfermedades Raras, Instituto de Salud Carlos III, 28029 Madrid, Spain; 3 Institute of Biomedical Research in Malaga (IBIMA), 29071 Malaga, Spain; 4 Spanish National Bioinformatics Institute (INB/ELIXIR-ES), Instituto de Salud Carlos III (ISCIII), 28020 Madrid, Spain; 5 Computational Systems Biology Group, Systems Biology Department, National Centre for Biotechnology (CNB-CSIC), 28049 Madrid, Spain; monica.chagoyen@cnb.csic.es

**Keywords:** biological network, network medicine, pathological phenotype, gene priorization

## Abstract

Network and systemic approaches to studying human pathologies are helping us to gain insight into the molecular mechanisms of and potential therapeutic interventions for human diseases, especially for complex diseases where large numbers of genes are involved. The complex human pathological landscape is traditionally partitioned into discrete “diseases”; however, that partition is sometimes problematic, as diseases are highly heterogeneous and can differ greatly from one patient to another. Moreover, for many pathological states, the set of symptoms (phenotypes) manifested by the patient is not enough to diagnose a particular disease. On the contrary, phenotypes, by definition, are directly observable and can be closer to the molecular basis of the pathology. These clinical phenotypes are also important for personalised medicine, as they can help stratify patients and design personalised interventions. For these reasons, network and systemic approaches to pathologies are gradually incorporating phenotypic information. This review covers the current landscape of phenotype-centred network approaches to study different aspects of human diseases.

## 1. Introduction

Living systems are characterised by a large number of components immersed in intricate networks of interactions, making them prototypical examples of complex systems. As such, many of their properties cannot be understood through the reductionist approach of molecular biology, which is based on the detailed characterization of individual molecular components, under the assumption that the properties of the system can be obtained from a simple combination of these. This approach fails to adequately reflect many aspects of living systems, which cannot be fully explained by a simple (additive) combination of their constituent molecular components [1,2,3,4]. In many cases, the properties of these components do not mean much outside the molecular level itself, and it is only in the context of the complex network of interactions and relationships with other components they acquire a biological meaning.

An alternative approach to biological phenomena from a systemic point of view, typically referred to as Systems Biology [5,6], complements the reductionist approach of Molecular Biology by studying living systems not from the point of view of their molecular components (“bottom-up”), but from higher levels of biological complexity (“top-down”). Frequently, these higher levels are represented by large and complex networks coding relationships between molecular components. Molecular networks are representations of relationships between different molecular components, with “relationship” often being defined in a broad sense. The discipline that mines these networks to extract biological knowledge is Network Biology [7,8]. Network Biology applies the tools of network analysis and graph theory to extract useful information from large molecular networks.

Although these systemic approaches are not new, they are experiencing a resurgence due to the ongoing revolution in “-omics” techniques, which allow one to obtain massive sets of relationships between molecular components that can be used to build molecular networks.

These systemic and network methodologies are not only being applied to basic research but to biotechnological and biomedical problems as well. Many pathologies cannot be reduced to a failure in a single gene or a small number of genes in a simple, additive way. These complex diseases are better reflected at the “network level”, allowing the integration of information on the relationships between genes, drugs, environmental factors and more. The discipline that approaches human pathologies from this systemic point of view is often called Network Medicine [9,10,11,12].

In medicine in general, and network medicine in particular, the basic unit for partitioning the complex human pathological landscape into discrete entities has been the “disease”. Nevertheless, this continuous landscape can also be partitioned into clinical signs, also known as symptoms or pathological phenotypes, which can be defined as the external manifestations of a pathological state (disease). These include entities such as fever, haemorrhage, inflammation, seizures, etc. Two unrelated diseases can share common phenotypes; conversely, two related diseases, or even the same disease manifested in different individuals, can show different sets of phenotypes. Although diseases were the basic units in the first network-based approaches to pathologies, network-medicine approaches focused on clinical phenotypes are becoming increasingly popular.

In this review, we provide an overview of these phenotype-centred network approaches to human diseases. We start with a short introduction to the main network concepts and general approaches used in network medicine, and continue with examples of applications to phenotypes. We also include a table with a summary of the main resources related this subject.

## 2. Overview of Network Approaches for Studying Human Pathologies

**Figure 1:**
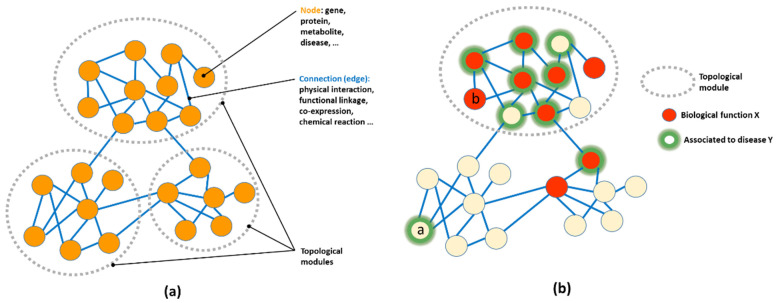
(**a**) Schematic representation of a generic biological network coding linkages between different molecular entities. This schematic network has three topological modules (clusters). Real biological networks can have tens of thousands of nodes and hundreds of thousands of relationships. (**b**) Relationships between topological, functional, and disease-related modules. The proteins involved in a specific function (“X”) are coloured red. Those associated with a given disease (e.g., those whose mutation is known to cause “disease Y”) are highlighted with green halos. Proteins known to be involved in “X” tend to cluster in the network. Those associated with disease “Y” tend to cluster in the same topological module, indicating that disease “Y” may be related to a malfunction of the biological process “X”. Alterations of other proteins in the same topological/functional module may also lead to the same disease as they disrupt the same process, even if they have not been detected yet (e.g., gene “b”). Conversely, genes far apart from the cluster might be discarded (e.g., “a”). Network propagation methods would tend to expand that initial set of Y-associated genes to the whole topological cluster as well as discard nonrelated genes.

**Figure 2:**
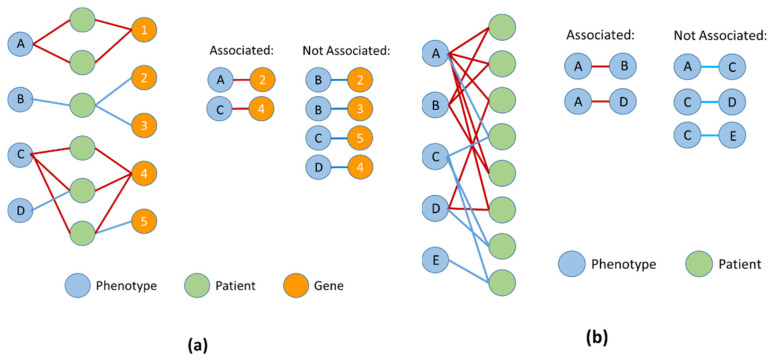
Overview of the use of multipartite networks to obtain phenotype–gene and phenotype–phenotype associations. (**a**) A phenotype–patient–gene tripartite network is constructed from patient data, such that a given phenotype in the cohort is linked to a gene in the cohort when there is at least one patient in the cohort who both manifests the phenotype and has a mutation that maps to the gene. Once the network has been built, it can be analysed to find those phenotype–gene pairs that are highly and specifically connected over many patients, using a statistical test to determine whether there is evidence that the pair is associated. There is also a third set of phenotype gene pairs, which are not connected via any patient (e.g., A-2), these are also considered not to be associated. (**b**) A phenotype–patient bipartite network is constructed from patient data, such that phenotypes are connected to patients when there is at least one patient in the cohort manifesting the phenotype. Once built, the network can be analysed to find pairs of associated phenotypes that are connected by many patients in a specific manner, using a statistical test. As with the phenotype–gene pairs, there are phenotype–phenotype pairs that are not connected by any patient (e.g., B–C). These must also be considered not associated.

A network (“Graph”, in mathematical terms) is just a representation of relationships between entities. Any phenomenon that can be represented as entities linked by relationships can be modelled as a graph. The entities are usually called nodes or vertices, and the relationships edges (Figure 1a). Both nodes and edges represent entities and relationships understood in the broadest possible sense. A node can represent a physical entity, such as a protein, gene, person, or even a computer, or other non-physical concepts, such as cell state, developmental stage, disease, or computer software. Similarly, edges can represent any type of generic relationship between nodes, such as physical interactions between proteins, chemical transformations between metabolites, hypertext links between two web pages, or subroutine calls in a computer program. Networks with more than one type of node or edge (e.g., some nodes representing proteins and other metabolites) are termed multipartite networks (e.g., Figure 2).

Network Medicine is largely concerned with the study of molecular networks, which represent generic relationships between molecular entities (Figure 1a). In general, these approaches try to extract disease-related information from the complex topological patterns present in these large networks.

A plethora of methodologies have been devised for extracting disease-related information from the topology of a molecular network. For more comprehensive reviews see [8,9,11]. A key concept in most of these approaches is that of the “disease-related module”. A module (also known as “cluster”) is a purely topological concept, and describes a set of network nodes that are enriched in internal connections (connections between themselves) compared to external connections (connections with other nodes of the network) (Figure 1). Topologically, they represent sub-networks that present some degree of independence from the rest of the network. In biological networks, these topological modules have been shown to represent functional modules, comprising functionally related sets of molecular entities [13]. For example, in protein interaction networks, it has been shown that they correspond to interacting proteins involved in the same biological process and/or forming a molecular complex (e.g., ribosome, proteasome) or working together in a signalling pathway [14,15,16]; in metabolic networks, they typically correspond to related metabolites involved in the same metabolic pathway [17]; in gene regulatory networks, they correspond to sets of related transcription factors involved in controlling a given cellular process [18]. Consequently, topological modules of biological networks can be considered functional modules (Figure 1b). Interestingly, it has also been shown that topological modules often represent disease-related modules, in the sense that genes associated with a given disease, when mapped into a biological network, tend to cluster together (e.g., [19,20,21,22,23]) (Figure 1b). In cancer, a complex disease characterised by a progressive accumulation of mutations, it has been shown that it is the concentration of mutated genes in network modules that characterises the transition from health to disease, rather than the general increase in the number of mutations [20]. Even in very complex diseases involving hundreds to thousands of genes, these tend to concentrate in a reduced number of modules/pathways (e.g., autism [24]). This relationship of topological modules with functional and disease-related modules forms the basis of most approaches in Network Medicine. This allows us to connect diseases with their underlying molecular mechanisms.

There are many approaches for locating these disease-related modules from an initial set of “seed” genes/proteins associated with a given disease [25,26]. These methods, usually termed “network propagation” or “network diffusion” approaches [26,27], detect the topological modules enriched in these seed genes (Figure 1b) using a variety of strategies. The seed genes are those known to be associated with the disease according to different pieces of evidence, such as phenotypic (e.g., disease-associated expression change) or genotypic data (mutations in affected individuals) [28].

Locating these disease-related modules is important for different reasons. On one hand, it allows the filtering of the original set of genes, discarding or adding new ones based on their belonging/closeness to the module (e.g., genes “a” and “b” respectively in Figure 1b). This is related to approaches for “gene priorisation” [29,30] that use network information, which are now routinely used for filtering, for example, the large set of variants showing up in “genome-wide association analysis” (GWAS) studies. It also allows one to predict new genes potentially associated with the disease, which could, for example, be more “druggable” (e.g., “b” in Figure 1b). Another advantage is that locating the modules associated with a disease allows it to be related to one or more biological functions (e.g., biological/metabolic pathways, macromolecular complexes), due to the relationship between topological modules and functional modules described earlier. This gives further insight into the cellular basis of the disease. Finally, as the network topology and connections represent different molecular mechanisms, having a picture of the network context of the disease-related genes allows us to better understand those mechanisms (e.g., which gene activates which other one in cascades of transcription regulation) and eventually design therapeutic interventions aimed at, for example, re-wiring a malfunctioning network (e.g., [31]).

Network approaches to human pathologies are not restricted to those molecular networks representing relationships between molecular entities. Many other disease-related data have been modelled as networks and studied from that point of view. These include drug–target relationships, drug–drug relationships, drug–disease associations, drug–side effect and disease–disease associations [32].

## 3. Phenotypes and Molecular Networks

To perform computational studies in general and systemic studies in particular on large collections of biomedical data, they have to be represented in a computer-tractable way, using standardised vocabularies (e.g., formal and standardised identifiers for genes, proteins) and ontologies (formal ways of representing knowledge, such as relationships between entities). In the case of phenotype data, there are multiple resources that provide these vocabularies and ontologies to describe different aspects of the human phenotypic landscape. A widely used vocabulary is that generated by the “Human Phenotype Ontology” (HPO) [33]. The HPO consists of a set of keywords describing human phenotypes related in a hierarchy, in which one can navigate from very general terms (e.g., “abnormality of the nervous system”) down to more specific ones (e.g., “seizure” → “non-motor seizure”).

Human diseases, characterised by a profile of distinctive HPO terms, can be clustered according to their phenotypic similarities. It has been known for a long time that many diseases and syndromes with similar phenotypes are caused by functionally related genes [21,34]. The extreme case is that of genetically heterogeneous diseases caused by genes involved in the same biological unit (such as a macromolecular complex, a pathway or process, or even an organelle). For example, the different types of Ehlers–Danlos syndromes are often caused by mutations in collagen genes or collagen interacting proteins that modify the structure, production or processing of collagen fibres. A large-scale study showed that genes causing the same (genetically heterogeneous) disease were frequently found to interact [35].

Beyond genetically heterogeneous diseases, several computational studies have observed that phenotypic similarity of otherwise distinct diseases is widely associated with shared protein interactions, both direct [36] as well as second-order interactions [37]. As mentioned above, diseases are organised in non-random modules in the human interactome, meaning that genes related to the same disease tend to be close in the interactome or even form a topological module [23]. The same modular organization observed for diseases has been quantified at the phenotype level [38], showing that genes associated with the same phenotype in genetic diseases tend to be close in the network of protein–protein interactions. Similarly, molecular-related phenotypes tend to overlap in the network, while unrelated phenotypes tend to be further apart.

## 4. Disease–Gene Predictions Using Phenotypic Descriptions and Molecular Networks

Early disease–gene prediction methods (e.g., [39,40]) were based on phenotypic descriptions of diseases, but did not use molecular networks. Direct protein interactions in human and model organisms were first used to predict novel genes associated with genetically heterogeneous diseases [41]. Lage et al. constructed a phenome–interactome network for human diseases, and used it to prioritise candidate genes [42]. In the absence at that time of curated phenotypic annotations such as those provided by the HPO, phenotypes were automatically extracted from OMIM records [43] using UMLS concepts [44]. UMLS defines a standardised vocabulary of medical concepts, and OMIM is a database of human genetic disorders. Since then, various studies have proposed a range of network-based methods to predict and prioritise novel disease-related genes [45], and several tools have been made available for this task (for a review see [30]).

Several approaches analyse a single molecular network (i.e., “homogeneous” network, with a single type of nodes and linkages). In these molecular network-based approaches, new genes are found in the proximity of known disease genes (“seed” genes). Algorithms that rely on global network distances, like random walk with restart (RWR) and network diffusion, have been shown to outperform those that use local distances [46]. Integrated functional networks provide better results than single-type interaction networks [47].

In the case of complex, oligogenic or genetically heterogeneous diseases, the seed is usually the set of already known disease genes [48]. However, how can we define seed genes for patients with rare conditions that have no clear clinical diagnosis? A possible strategy is to compile seed genes from conditions with clinical manifestations (phenotypes) similar to those observed in the patient [27,49,50]. Using this strategy, methods that analyses the output of genome-wide sequencing platforms, such as Exomiser [51] and Phen-Gene [52], allow researchers to score candidate genes using a network-based approach. In these approaches, the clinical manifestations of patients should be provided using a controlled vocabulary, such as the HPO. These manifestations are then automatically compared to those of known diseases to construct a set of seed genes to start the network analysis. Several disease similarity metrics can be used to find phenotypically similar diseases (for a recent review see [53]).

Instead of using a single molecular network and establishing initial probabilities for seed genes based on phenotypic similarities, alternative approaches analyse multipartite (heterogeneous) networks. RWRH (random walk with restart on heterogeneous network) combines a disease–disease network (constructed based on phenotypic similarity) with the molecular network [54]. These two networks are linked by the connections between diseases and molecular nodes, generating a bipartite network. This approach was shown to outperform the equivalent method using a single molecular network in the task of assigning genes to diseases.

Disease–phenotype associations can be explicitly modelled as a bipartite network. This disease–phenotype network, together with parenthood relationships between phenotypes, a molecular network, and the corresponding inter-network links, establishes a tripartite network (diseases, phenotypes, and genes). This tripartite network, or context-sensitive network (CSN) has been shown to outperform RWRH in assigning genes to their corresponding diseases [55].

## 5. Phenotype–Gene Predictions

Similar methods to those described above aimed at predicting disease-related genes using molecular networks can be used to associate genes with phenotypes. The inverse problem, the prediction of phenotypes for a given gene/protein is outside the scope of this review (see CAFA challenge [56]).

Li et al. integrated protein interactions as well as disease and phenotype information to predict novel genes associated with phenotypes [57]. Their motivation was the study of the molecular mechanisms underlying phenotypes, which are the basis for personalised diagnosis and treatment in traditional Chinese medicine. Kahanda et al. used protein–protein interactions combined with other types of data, such as functional annotation and literature to predict phenotype–gene associations [58]. Another approach for assigning genes to phenotypes is that developed by Petegrosso et al., who used a transfer learning approach (tlDLP) on a tripartite network that contains HPO terms (and their parenthood relationships), Gene Ontology (GO) terms representing functional annotations (together with their ontological relationships) and a molecular network (protein–protein interactions) [59]. Gonzalez-Perez et al. [60] compared an RWR approach to a connectivity significance approach [19] using a single molecular network, obtaining overall better results with RWR. Yang et al. used a multipartite network embedding algorithm (LSGER) [61]. They obtained an overall improvement compared to FSGER (Fisher-based statistical model, as a baseline method) and PRINCE [27].

It is worth mentioning that the accuracy of phenotype–gene predictions depends not only on the method used, but also on several biomedical factors. This was shown in a recent study [60] in which the performance of RWR to predict genes associated with phenotypes was assessed and related to aspects like disease onset and pace of progression, phenotype prevalence, and gene product function. The biomedical factors that most affected prediction performance were the monogenic or oligogenic nature of the disease, the type of phenotype, and the mode of inheritance. Better predictions were obtained for genes involved in oligogenic diseases (in contrast to monogenic diseases). Neoplasm phenotypes and abnormalities of the blood were among the best predicted phenotypes, in contrast to abnormalities of the eye or the nervous system that were poorly predicted. Phenotype–genes of autosomal-dominant inherited diseases obtained better global results than those with autosomal recessive inheritance.

There are other ways to associate genes with phenotypes that involve networks, although not necessarily molecular networks. For example, PhenFun [62] connects phenotypes with genes based on a cohort of patients with genomic disorders. This is achieved by identifying the genomic regions shared by multiple patients and connecting the genes that map to these regions to the phenotypes exhibited by these patients. Then, those phenotypes that are connected to the same gene via multiple patients are considered potentially associated. The strength and significance of the association is assessed using statistical methodology, as illustrated in Figure 2a and described in detail in [62]. They also used the genes associated with each phenotype to look for functional enrichment in terms of biological pathways. This was based on the assumption that it might be a specific pathway that leads to the phenotype, and alterations in different genes from the same pathway might lead to the same effects.

## 6. Phenotypes and Patient Stratification

One of the overarching themes of clinical research in the last decade has been the recognition that a one-size-fits-all disease label is often insufficient. It has been known for a long time that common diseases such as asthma are really composed of many subgroups or endotypes, characterised by differences in term of manifested phenotypes as well as often distinct underlying mechanisms [63]. A powerful way to discover subgroups within a disease is by stratifying patients. This can be done based purely on molecular data, such as using genetics and “omics” datasets to build gene signatures and model the underlying processes [64,65].

However, much can be achieved using phenotypic data. Patients should be well phenotyped, both in terms of breadth (ensuring all aspects of the patient’s disease is covered) and also depth (ensuring that the description of the disease is suitably precise). Most of the methods used to cluster patients are unsupervised approaches such as hierarchical clustering or related methods such as Partitioning Around Medoids (PAM), applied to a matrix of patients and factors.

It should be made clear that terminology is important here—there are studies that classify patients into different groups which they refer to as “phenotypes”, classifying based on factors such as levels of serum proteins [66] and scores on clinical tests [67]. In these studies, they are often talking about a small number of phenotypes. Conversely, in other work, phenotypes (such as HPO terms assigned to patients) are considered the factors themselves, and are used to perform clustering of the patients into groups.

In recent work, researchers looked in detail at how to assess phenotyping breadth and depth in a patient cohort [68]. Patients from three cohorts with very different properties were clustered based on their phenotypic profiles. For the cohorts with more inconsistent and less informative phenotypic data, it was found that many patients ended up in uninformative clusters, which included only a small number of very broad and unspecific phenotypes such as “intellectual disability”. Clearly, such broad groups are not sufficient or informative for stratifying patients in a useful manner, and it was suggested to remove uninformative patients from the dataset, given that filtering patients based on very minimal criteria (included patients must have more than two pathological phenotypes assigned) greatly changes the properties of the dataset and the grouping. Of course, if possible, further clinical examination can also improve the information available in patient phenotypic profiles [68].

Various methods exist for this purpose. Work in the field of pain has long used quantitative sensory testing (QST) for patient testing [69]; however, its utility is often questioned [70]. In recent work from Vollert et al., QST sensory profiles were used to stratify patients into one of three groups, “sensory loss” “thermal hyperalgesia” or “mechanical hyperalgesia” [67], using a deterministic algorithm based on features in their QST profile. Moreover, they also suggested a probabilistic algorithm that allowed a patient to potentially be assigned to more than one group.

Combining phenotype data with genetic data allows us to better understand the reasons for the stratification of patients. In seminal work from the BRIDGE-BPD consortium, looking at heritable bleeding and platelet disorders (BPD), Westbury et al. performed patient clustering on cohort members based on HPO data [71]. They found that the patient groups formed by the clustering corresponded to suspected BPD syndromes and pedigree membership. Patients with variants in specific genes also tended to cluster together, showing that patient stratification cannot only tell us how patients are related, but perhaps why, i.e., the underlying molecular/genetic mechanisms.

There has been increased interest in this area over recent years due to the COVID-19 pandemic. In a new and exciting study Mueller et al. used immune phenotypes for patient stratification [66]. More specifically, they measured serum cytokine levels for each patient and then performed clustering on the data, identifying three major groups of patients. Interestingly, group membership was highly correlated with the results of certain clinical tests.

Bringing together personalised medicine and molecular networks, there have also been several papers that perform network analysis at the individual level. Work by Liu et al. used expression data and protein interactions from the STRING database [72] to build sample-specific networks [73]. Other studies have focused on transcriptomic data, using linear interpolation in an attempt to reverse engineer single sample networks [74]. These personalised network approaches are set to improve greatly, as we move from bulk RNA sequencing to single-cell RNA sequencing, allowing a huge amount of information to be obtained at the individual level. Already, methods are being developed to analyse such data and produce networks down to the cellular level [75].

## 7. Phenotypes and Co-Morbidity

As well as stratifying patients, phenotype data from patient cohorts and disease databases can be used to identify patterns of co-dependency and comorbidity between phenotypes. There are methods to achieve this-based directly on phenotype co-occurrence within patient cohorts, or across many thousands or even millions of patients based on hospital records.

Many methods based on patient data use International Statistical Classification of Diseases and Related Health Problems (ICD) codes [76]. For example, in pioneering work, Hidalgo et al. used ICD-9 codes from thirteen million patients to identify comorbid disease relationships [77]. For each pair of diseases, they calculated relative risk of both being affected in the same patient to quantify comorbidity (Pearson’s correlation coefficient was also used). The pair information was then used to construct a Phenotypic Disease Network (PDN), which showed phenotypes belonging to specific disease families tending to be close in the PDN. This work has been heavily cited, and similar approaches have been applied to a range of other diseases, including ischemic heart disease [78], in which a PDN of comorbid phenotypes was built for sufferers of this disease. It has also been recently applied to look at comorbidity in depression [79], for which the authors used Pearson’s correlation to measure comorbidity between a large spectrum of phenotypic conditions. Both works used ICD-10 codes. Comorbidity networks can also be built between ICD-10 derived diseases. Such a method has been applied to type-2 diabetes, for example [80]. Other work has leveraged the relative risk calculation alongside temporal data to build disease progression networks, with clear potential use for diagnosis and prognosis [81].

ICD codes are widely used for creating hospital bills and insurance claims and as such are highly used and readily available. While ICD codes have the advantage of allowing the analysis of very large numbers of patients, the system has been criticised, largely due to potential bias related to their use in billing and other related administrative tasks [82]. The “human phenotype ontology” (HPO) described earlier provides an alternative system for assigning phenotypes, which due to its tree-like structure and other attributes, is well suited to comorbidity analysis. To this end, the PhenCo methodology was developed [83], which takes patient cohort data, consisting of phenotypic profiles for patients, annotated using the HPO. These profiles are then deconstructed to produce a bipartite network linking patients and profiles, such that it is possible to identify pairs of phenotypes that are shared by many patients (Figure 2b). Moreover, these pairs of phenotypes can be scored, such that those pairs that tend to occur together more often are scored highly.

These phenotype pairs by themselves are of interest as they help better understand phenotypic patterns within the cohort and formally quantify clinicians’ intuitions of how certain phenotypes tend to manifest together within their cohort. However, the real power of the method occurs when the pairs are combined to produce a phenotype network. PhenCo not only builds such a network, but implements an edge-based clustering method to obtain clusters of phenotypes that tend to occur together. Furthermore, it can integrate the phenotypic data with genomic data, such that phenotypes can be associated with functional systems, in a similar manner to the previously described PhenFun system [62]. This allows identification of clusters of phenotypes that overlap together, as well as clusters where the component phenotypes map to the same functional systems, providing insight into the molecular cause of the clustered phenotypes (Figure 2a).

There are also approaches that use HPO terms alongside data obtained directly from disease databases such as OMIM [43] and Orphanet [84]. For example, a recent study using the PhenoClusters methodology [85] gave insights into neuromuscular disease (NMD). The method obtains NMD related diseases from OMIM using keyword searching and expert manual curation. It then extracts NMD-related phenotypes by looking for HPO terms that are overrepresented within the NMD-related diseases. These are used to build a bipartite network, connecting phenotypes with diseases, and in a manner analogous to the PhenCo method described above, to find phenotypes that tend to occur together across diseases. These phenotype pairs are scored and can be assembled together to build networks that can in turn be clustered. Moreover, known disease–gene association data can be used to find clusters of related phenotypes that are associated with genes involved in similar pathways. Through collaboration with experts in the field of NMDs, it was possible to identify important clusters that can aid in differential diagnosis [85].

There are additional methods that, while not measuring comorbidity directly, attempt to look for relationships between phenotypes by incorporating additional data sources. In a study described earlier [38], similarities between phenotypes were quantified using the properties of the interactome. Similar work to this study combined HPO phenotypes with protein–protein interaction data, by mapping phenotypes to the interactome, and calculating similarity between the modules formed within the interactome by the distinct phenotypes [86].

As well as HPO terms and ICD codes, exciting approaches have also used symptom terms in the Medical Subject Headings (MeSH) [87] metadata fields of PubMed entries to build a human symptoms–disease network [37]. This idea has been extended to produce a tool that is capable of retrieving from the scientific literature relationships between HPO terms and terms from other annotation systems, including Gene Ontology as well as different biomedical ontologies [88]. However, this method differs in terms of the underlying approach, in that it searches for the terms within the abstracts of PubMed articles, rather than relying on the MeSH fields.

## 8. Resources

Most of the resources mentioned (e.g., databases and software tools) are free for academics and can be accessed on-line. In this section, we include a table with a summary of the main resources commented in this review as well as others related to the subject, including their web addressed (Table 1).

## 9. Discussion

Molecular biology revolutionised medicine by providing insight into the molecular mechanisms underlying pathological processes. The diagnosis and the treatment of many human diseases changed as molecular entities (e.g., genes, proteins, metabolites) started to be used as markers of a disease or targets to cure it. This molecular approach is highly “reductionist”, in the sense that a single, or a very small number of molecular entities, is assumed to be the cause (and target for an eventual treatment) of a disease. That assumption, fundamentally correct for some pathologies (e.g., monogenic diseases), has problems when applied to many complex pathologies such as cancer or Alzheimer’s. To complement these reductionist approaches, systems- and network-biology methods are being applied to the study of human pathologies, in what sometimes is called “Network Medicine”. The change of paradigm in these new approaches is that, due to their complexity, most diseases are better reflected at the level of complex networks, instead of single genes. Even in “classic” monogenic diseases, the causative gene is immersed in complex networks and hence, even if the onset of the disease depends on that single gene, other important factors, such as its severity or patient-specific manifestations depend on many other genes/mutations, requiring a more systemic approach for understanding them (e.g., cystic fibrosis [90]). From this point of view, diseases are seen as emergent properties of complex networks, which are affected by both genetic and environmental factors, and as perturbations in the network structure (e.g., re-wiring) more than in the nodes (genes) themselves [8].

Approaching pathologies from a phenotypic point of view has some advantages with respect to the traditional disease-based point of view. Clinical signs can be closer to the molecular mechanism(s) behind the pathology, as they are more directly related to the phenotypic manifestations. Moreover, by definition, clinical signs are directly observable, and in many cases even quantifiable, and hence they can be reported for any pathological condition. Sometimes a particular disease cannot be diagnosed and only a collection of symptoms is reported. Clinical signs are also key for personalised medicine, as the patient symptom profiles can help in their stratification and the design of personalised interventions. For these reasons, network and systemic approaches to diseases are also gradually adopting this point of view and incorporating phenotypic information.

To incorporate phenotypic information, controlled vocabularies and ontologies for formally representing that kind of data are required, such as that developed by the HPO and other resources such as IDC or MeSH. Similarly, new methodologies for massively extracting phenotypic information from un-structured resources and representing them using those ontologies are also important (e.g., CoMent [88]). This would allow us to identify pathological phenotypes that tend to occur together across potentially millions of patients, in a similar manner to the previously mentioned methods PhenCo and PhenoClusters. Similarly, tools such as CoMent could potentially be extended to obtain co-occurrent phenotypes, as such pairs of phenotypes that tend to be mentioned together or not at all may represent potentially comorbid phenotypes.

To conclude, it is clear that the ongoing revolution in the generation of biological data, improvements in computational techniques for data analysis, particularly network analysis, and the ever-increasing recognition of the importance of deep phenotyping of patients [91], are providing us with a wealth of potential for better understanding disease and eventually finding better treatments.

## Figures and Tables

**Table 1:** Main online resources related to network approaches to diseases and phenotypes.

Name	Description	URL ^1^	Reference
CytoScape	Widely used software for interactively representing and studying biological networks. Freely available for different operative systems	https://cytoscape.org/	[89]
STRING	Resource with networks of interactions and functional relationships between proteins in different organisms, inferred from different evidences	https://string-db.org/	[72]
Human Phenotype Ontology (HPO)	Controlled structured vocabulary for describing different aspects of human disease phenotypes/clinical signs	https://hpo.jax.org/app/	[33]
Online Mendelian Inheritance in Man (OMIM)	Catalogue of human genetic disorders and their related genes	https://www.omim.org/	[43]
Orphanet	Resource with information on rare diseases and orphan drugs	https://www.orpha.net/	[84]
Medical Subject Headings (MeSH)	Controlled vocabulary used to annotate PubMed bibliographic entries	https://www.ncbi.nlm.nih.gov/mesh/	[87]
CoMent	Relationships between biomedical concepts extracted from the literature	https://sysbiol.cnb.csic.es/CoMent/	[88]

^1^ All URLs accessed on January 2022.
